# Chronic Atrophic Gastritis with Negative Intrinsic Factor and Parietal Cell Antibody Presenting as a Severe Hemolytic Anemia

**DOI:** 10.1155/2020/8697493

**Published:** 2020-05-15

**Authors:** G. F. Cittolin-Santos, S. Khalil, J. K. Bakos, K. Baker

**Affiliations:** Medical University of South Carolina, Department of Internal Medicine, Charleston, SC, USA

## Abstract

A 28-year-old Caucasian male with Hashimoto's disease and vitiligo presented with two weeks of dizziness on exertion following pharyngitis which was treated with prednisone 40 mg by mouth once a day for five days. Initial workup revealed anemia, elevated lactate dehydrogenase (LDH), and low haptoglobin. He underwent workup for causes of hemolytic anemia which was remarkable for a peripheral blood smear with hypersegmented neutrophils and low vitamin B12 levels concerning for pernicious anemia. Parietal cell and intrinsic factor antibodies were negative, and he then underwent an esophagogastroduodenoscopy with biopsy. The biopsy was negative for *Helicobacter pylori*, and the immunohistochemical stains were suggestive of chronic atrophic gastritis. He was started on vitamin B12 1,000 mcg intramuscular injections daily. His hemoglobin, LDH, and haptoglobin normalized. Given the absence of the parietal cell antibody and intrinsic factor antibody, this is a rare case of seronegative pernicious anemia.

## 1. Introduction

Pernicious anemia is often associated with other autoimmune diseases such as Hashimoto's disease and vitiligo [1]. Severe cases of vitamin B12 deficiency can cause ineffective erythropoiesis and a hemolytic anemia [2–5]. The differential and workup for a hemolytic anemia is broad and can be clouded by concomitant infections and other autoimmune conditions. Historically, the Schilling test was used to diagnose pernicious anemia; however, advances in testing have led to the measurement of intrinsic factor level, antiparietal cell antibody, anti-intrinsic factor antibody, and histologic analysis [6, 7]. Here, we report a case of hemolytic anemia and ineffective erythropoiesis associated with atrophic gastritis due to seronegative atrophic gastritis causing pernicious anemia.

## 2. Case Presentation

**Figure 1:**
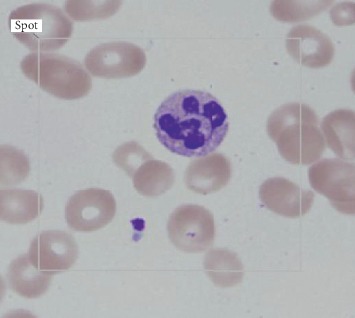
Peripheral smear with hypersegmented neutrophils.

A 28-year-old male with a past medical history of Hashimoto's disease and vitiligo presented with two weeks of exertional dizziness, fatigue, nausea, pallor, and an intermittent generalized maculopapular rash. He denied fevers, chills, joint pain, easy bruising, recent travel, or sick contacts. He had also been recently treated for pharyngitis by his primary care physician with prednisone 40 mg by mouth once a day for five days due to the severe throat pain with improvement of symptoms. He denied any prior personal or family history of blood disorders. He denied any new medications other than prednisone use for his pharyngitis. His only medication was levothyroxine 150 mcg by mouth once a day. On admission, his temperature was 37.2°C, pulse 102 bpm, blood pressure 119/81 mmHg, respiratory rate 16, and oxygen saturation 100% while breathing ambient air. He had pale conjunctiva and skin without bruising, petechiae, or rashes. He had splenomegaly, but the remainder of the physical examination was normal. Initial workup was remarkable for macrocytic anemia, elevated LDH, low haptoglobin, and undetectable vitamin B12 levels (Table 1). An infectious workup and serologic workup for autoimmune anemia and pernicious anemia were also performed (Table 2). Patient had normal hemoglobin electrophoresis. Initially, flow cytometry testing for paroxysmal nocturnal hemoglobinuria (PNH) was not performed, while the DAT results were pending, although PNH was a diagnosis on the differential. Peripheral smear was notable for hypersegmented neutrophils, macrocytic normochromic anemia, thrombocytopenia, and lacked schistocytes (Figure 1). Abdominal ultrasound was significant for splenomegaly.

**Figure 2:**
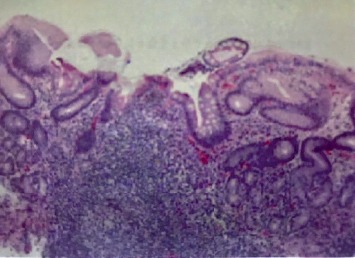
Gastric biopsy showing lymphoplasmacytic cells in the lamina propria of gastric tissue. No neutrophilic activity is identified. Atrophy was noted without dysplastic alterations.

The hypersegmented neutrophils in the peripheral smear without schistocytes, the laboratory findings of hemolytic anemia, and the undetectable B12 levels were all consistent with the diagnosis of pernicious anemia. The patient was started on intramuscular vitamin B12 1,000 mcg on day three of admission. On the second and fourth day of hospitalization, the patient's hemoglobin was 5.2 and 6.2 g/dL, so the patient was transfused one unit of packed red blood cells (pRBCs) each day with a hemoglobin response to 6.5 and 7.1 g/dL, respectively. On hospital day eight, his hemoglobin improved without transfusion to 7.5 g/dL, so he was deemed stable for discharge with close follow-up. The patient underwent regular B12 replacement—weekly for a month and then monthly thereafter. He then underwent an outpatient esophagogastroduodenoscopy (EGD), which demonstrated widespread atrophy of the stomach. Biopsies were taken of the mucosa that suggested chronic atrophic gastritis with histologic and immunohistochemical findings consistent with pernicious anemia without evidence of *Helicobacter pylori* (Figure 2). The patient was continued on B12 replacement and presented for outpatient follow-up on day 15, 25, and 60 after his initial hospital presentation with hemoglobin level 10.7, 12.5, and 14.5, respectively. He had no further evidence of hemolysis and did not require any further intervention other than the B12 injections. This is an unusual case of hemolytic anemia from ineffective erythropoiesis secondary to seronegative pernicious anemia and B12 deficiency.

## 3. Discussion

Identifying the culprit for an acute episode of anemia can be challenging. There are multiple etiologies of acute anemia including hemolysis, and the cause of hemolysis can vary greatly [8, 9]. Anemia due to B12 deficiency is not usually associated with hemolysis, and it is not classified as a hemolytic anemia [10, 11]. Despite that, it is important to consider B12 deficiency as the cause of hemolysis when faced with an increase in LDH levels higher than 5–10 times the upper normal limit, especially with accompanying cytopenia. Although uncommon, B12 deficiency causes one of the highest peaks in LDH due to the ineffective erythropoiesis and premature RBC death. Paroxysmal nocturnal hemoglobinuria also causes a marked LDH peak and sometimes is associated with cytopenias and should be considered in this setting as well [9]. There are well-known algorithms for the workup of anemia; however, standard algorithms do not always apply and can be obscured by confounding diseases. As previously described (Barcellini and Fattizzo, 2015), the utilization of clinical and hemolytic markers is helpful in diagnosing hemolytic anemias [9]. Reaching the correct diagnosis is important because each condition requires specific treatment and follow-up.

We faced a case of acute hemolytic anemia in a patient with known autoimmune disease. It is known that patients with autoimmune conditions are especially prone to develop autoimmune hemolytic anemia [5]. However, a majority of patients with pernicious anemia present with subacute to chronic symptoms [12], and the case above is an atypical presentation. Although B12 deficiency has been previously associated with intramedullary hemolysis and ineffective erythropoiesis, hemolysis due to B12 deficiency is rare. We also considered PNH as a possible cause due to the markedly elevated LDH levels. However, the patient had improvement with B12 treatment when the final DAT testing resulted, so we did not order a flow cytometry testing for PNH due to the low likelihood of this diagnosis at that time. Furthermore, the sensitivity of antiparietal antibodies is high, and it is uncommon for both antiparietal and anti-intrinsic factor antibodies to be negative. It has been shown that these antibodies are present in ninety percent of patients with pernicious anemia. Seronegativity may be explained by complete antibody-to-antigen binding so that no free antigen is circulating by antibody production failure or by the disappearance of the antibody due to antigen disappearance. In our case, the recent course of prednisone may have altered the antibody response. Type-I auto-antibodies that block the binding of the intrinsic factor and vitamin B12 were only demonstrated in approximately seventy percent of patients with pernicious anemia. Type-II auto-antibodies that bind to another site separate from the vitamin B12-binding site are also only present in approximately thirty-five to forty percent of these patients [13]. This explains how patients with pernicious anemia may have seronegative findings.

Hypersegmented neutrophils on the peripheral smear were compatible with B12 deficiency (Figure 1). The paucity of schistocytes in the peripheral smear leads us to believe that the hemolytic process was intramedullary, a phenomenon which has been previously described in cases of extreme B12 deficiency. Also, marked intravascular hemolysis is usually associated with dark “brownish” urine discoloration due to the presence of hemosiderin bound to iron in the urine, which was not present in this case [9, 14]. We observed a response in reticulocyte count and Hgb levels around seven days after starting intramuscular vitamin B12 1,000 mcg daily injections. Besides a two-time pRBC transfusion and daily vitamin B12 injections, the patient did not require any other interventions, and his symptoms resolved shortly thereafter. The patient underwent an outpatient EGD with gastric biopsy following discharge that confirmed the presumptive diagnosis by demonstrating atrophy with lymphoplasmacytic cells in the lamina propria of gastric tissue similar to atrophy and diffuse lymphoplasmacytic cells seen in a typical case of pernicious anemia (Figure 2).

This case is an atypical presentation of B12 deficiency due to the acute onset of severe symptomatic anemia and the negative serologic workup for pernicious anemia. However, the patient's resolution in anemia and hemolysis following vitamin B12 injections along with the biopsy result of chronic atrophic gastritis confirmed the diagnosis of seronegative pernicious anemia. Anemia due to B12 deficiency is often successfully treated with supplementation without even evaluating serology, but having a confirmatory test diagnosing pernicious anemia is important due to possible complications if the condition is left untreated. Patients with untreated pernicious anemia may develop neurological symptoms that range from paresthesia to ataxia, generating a clinical scenario of combined sclerosis of the spinal cord that may lead to irreversible sequelae. Also, the autoimmune gastritis of pernicious anemia is associated with susceptibility of gastric tumors—carcinoid, carcinomas, and non-Hodgkin's malignant lymphoma. Thus, surveillance of gastric tumors is warranted, and the patients should be referred to a gastroenterologist [15]. This case illustrates why one should keep pernicious anemia in the differential diagnosis for hemolytic anemias, especially when LDH levels are exceedingly high and associated with cytopenias, despite negative antiparietal cell or intrinsic factor antibodies.

## Figures and Tables

**Table 1:** Laboratory data.

Variable	Reference range	Day 1	Day 2^*∗*^	Day 3^*∗∗*^	Day 4^*∗*^	Day 5	Day 6	Day 7	Day 8	Day 15	Day 22
Hematocrit (%)	41–53	16.2	14.2	15.9	18	17.5	20.9	21.9	24	32.6	38.2
HB (g/dL)	12–18	6.1	5.2	6.5	6.2	7.1	7.1	7	7.5	10.7	12.5
Retic index		0.29				0.15	0.21	3.61	5.28	2.26	
Platelets (K/*μ*L)	140–440	94	90	65	73	62	70	82	66	383	252
MCV (fL)	77–106	109									
WBC (K/CUMM)	4.8–10.8	5.6	5.35	4.47	4.48	5.67	6.51	5.04	4.24	6.7	5.6
LDH (U/L)	100–240	6941					6170	4048	3487		
AST (U/)	10–36	270	191	189			172	109	71		
ALT (U/L)	12–78	163	127	128			170	169	71		
Fibrinogen (mg/dL)	231–486	264									
Total bilirubin (mg/dL)	0.2–1.3	1.4	1.4	2.3			1.2	0.9	1		
Haptoglobin (mg/dL)	14–258	<8					<8				23
Serum iron (mg/dL)	50–175	163									
Ferritin (mg/mL)	21.8–322	662									
TIBC (mcg/dL)	245–425	182									
G6PD (U/g Hb)	9.9–16.6	14.2									
Folate (ng/ml)	7–31.4	12.9									
Vit B12 (pg/mL)	211–911	<146				>1700					991
Erythropoietin (mIU/mL)	2.6–18.5	114									
TSH (mIU/mL)	0.35–4.94	4.56									

G6PD = glucose-6-phosphate dehydrogenase; TSH = thyroid-stimulating hormone; ^*∗*^one unit of packet RBC (pRBC) was transfused that day; ^*∗∗*^daily B12 injections were started that day.

**Table 2:** Infectious and autoimmune workup.

CMV PCR	Negative
EBV PCR	Negative
HIV PCR	Negative
Hepatitis C antibody	Negative
Coombs test	Negative
Parietal cell antibody	Negative
Intrinsic factor antibody	Negative
Cryoglobulin	Trace after 24 h
Direct antiglobulin test^*∗*^	Negative

^*∗*^Direct antiglobulin test (DAT) includes DAT polyspecific, immunoglobulin G (IgG), complement 3/complement 3d antibodies, 4C low ionic strength saline (LISS) wash antibodies, polyethylene glycol antibodies, IgG and IgA gel antibodies, and acid eluate.
